# Hairy Polyp Presenting With Respiratory Distress in an Infant With Cleft Palate

**DOI:** 10.7759/cureus.71489

**Published:** 2024-10-14

**Authors:** Yuji Fujita, Yurie Takise, Yuni Masuyama, Itsuo Nakajima, Yumi Nozawa, Kazuyuki Ishida, Shigemi Yoshihara

**Affiliations:** 1 Pediatrics, Dokkyo Medical University, Mibu, JPN; 2 Otorhinolaryngology - Head and Neck Surgery, Dokkyo Medical University, Mibu, JPN; 3 Diagnostic Pathology, Dokkyo Medical University, Mibu, JPN

**Keywords:** apnea, hair polyp, polypoid tumor, poor feeding, respiratory distress

## Abstract

Hairy polyps often develop from the upper respiratory tract. They cause various symptoms, such as respiratory distress. A one-month-old boy with a cleft palate was referred to our hospital due to feeding difficulty, stridor, and labored breathing. Physical examination revealed suprasternal retractions and apnea. A laryngoscopy was performed due to suspicion of upper airway obstruction, and a pedunculated, mobile mass occupying the pharynx was observed. Surgical resection was performed perorally. Histopathological examination confirmed the diagnosis of a hairy polyp. The most common complaint of hairy polyps at the time of admission was dyspnea. It has been reported that hairy polyps <3 cm in diameter is often overlooked during oral examinations. However, respiratory distress and cardiac arrest are more common in these polyps than in those measuring >3 cm. Approximately 10% of patients with hairy polyps reportedly have a cleft palate, which may be related to various arch deformities such as cleft palate. Hairy polyps should be considered a cause of respiratory distress in neonates and infants, especially those with a cleft palate.

## Introduction

Hairy polyps are considered to be likely developmental abnormalities of the first or second branchial cleft, which often develop from the upper respiratory tract, such as the posterior wall of the nasopharynx, the upper surface of the soft palate, and palatine tonsils. They cause various symptoms, such as feeding disorders and respiratory distress [1,2]. Herein, we describe an infantile case with a cleft palate who presented with poor feeding and respiratory distress and was subsequently diagnosed with a hairy polyp.

## Case presentation

A one-month-old boy with a cleft palate was referred to our hospital due to feeding difficulty, stridor, and labored breathing. He was diagnosed with cleft palate after birth. His mother noticed a tumor in the mouth at home and consulted a physician when the patient was 14 days old. Decreased milk intake was observed since the patient was 27 days old. When the patient was 31 days old, he began choking while feeding and had a poor complexion, as well as respiratory distress. He was subsequently admitted to our hospital, and a tumor was found on the posterior pharyngeal wall. His vital signs included a body temperature of 36.8°C, a blood pressure of 80/62 mmHg, a heart rate of 147 beats per minute, and oxygen saturation of 92-95%. His body weight was 3,035 g (birth weight, 2,700 g). Physical examination revealed suprasternal sunken breathing and apnea. Laboratory examination revealed no abnormal findings (Table 1).

**Table 1:** Laboratory data of the patient A blood examination revealed no abnormal findings. The values in parentheses indicate the normal ranges for each parameter. ALT: alanine aminotransferase, AST: aspartate aminotransferase, BUN: blood urea nitrogen, CRP: C-reactive protein, LD: lactate dehydrogenase, WBC: white blood cell

Hematology			Biochemistry		
Complete blood count			AST	21 (10-40)	U/L
WBC	7,400 (3,900-9,800)	/µL	ALT	14 (5-40)	U/L
Neutrophil	26.6 (40-74)	%	LD	217 (124-222)	U/L
Eosinophil	1.9 (0-6)	%	Albumin	4.0 (3.8-5.2)	g/dL
Lymphocyte	66.7 (18-59)	%	BUN	7.7 (8-22)	mg/dL
Hemoglobin	14.7 (13.5-17.6)	g/dL	Creatinine	0.18 (0.61-1.04)	mg/dL
Platelet	32 (13.1-36.2)	×10^4^/µL	CRP	<0.01 (<0.14)	mg/dL

A laryngoscopy was performed due to suspicion of upper airway obstruction, and a pedunculated, mobile mass occupying the pharynx was observed (Video 1).

**Video 1:** Laryngoscopy findings Laryngoscopy shows a pedunculated, mobile mass lesion occupying the pharynx.

**Figure 1:**
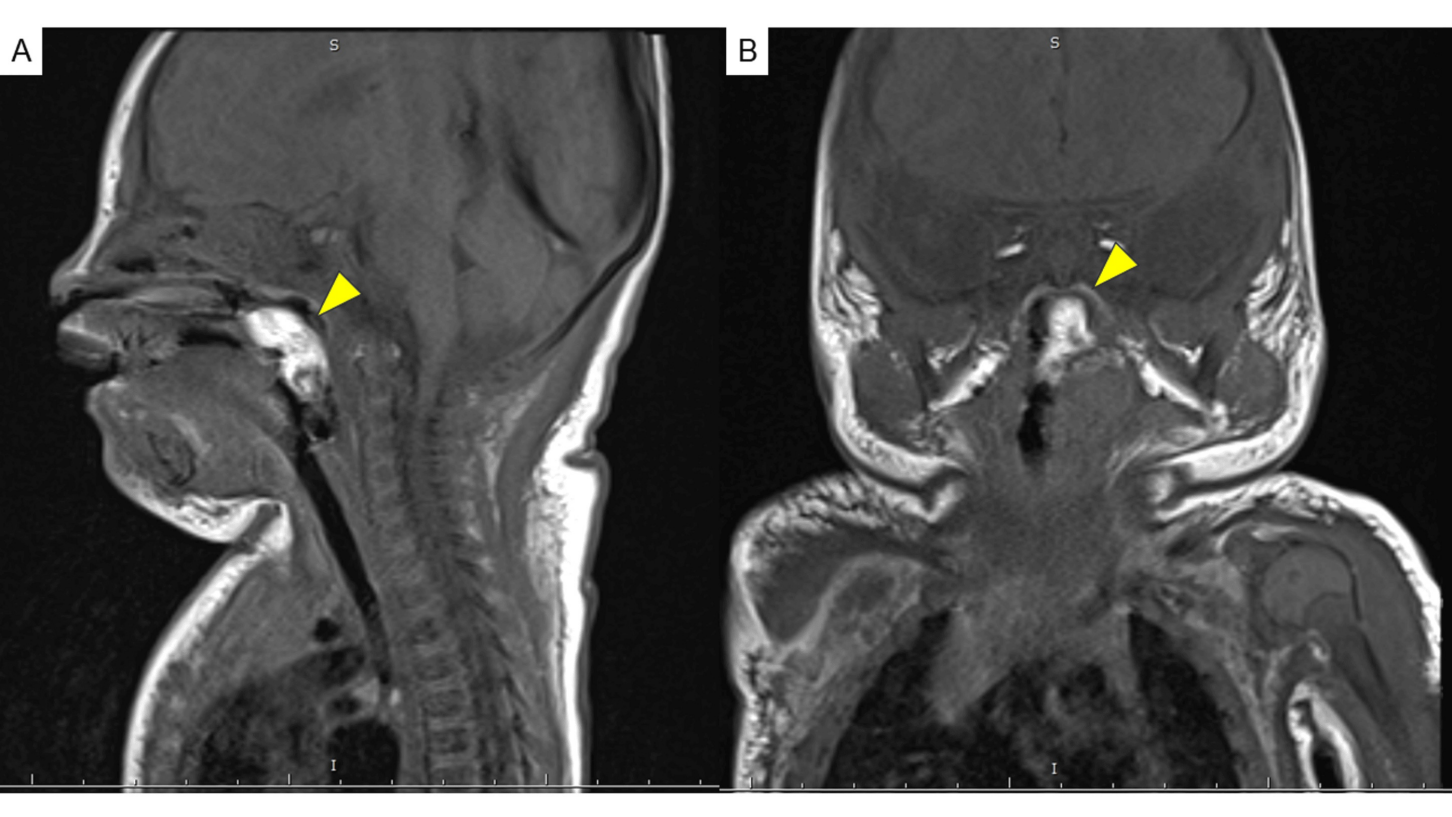
Magnetic resonance images of the neck The images reveal a mass (arrow) near the left uvula, surrounded by fatty components that showed high signal intensity on T1-weighted imaging, the central area of which is equivalent to that of a muscle with a pedicle (A, sagittal view; B, coronal view).

Tracheal intubation was performed due to frequent apnea attacks. Magnetic resonance imaging of the neck revealed a mass near the left uvula surrounded by fatty components that showed high signal intensity on the T1-weighted image. The central area of the lesion was equivalent to that of a muscle with a pedicle, which raised suspicion for a hairy polyp (Figure 1).

**Figure 2:**
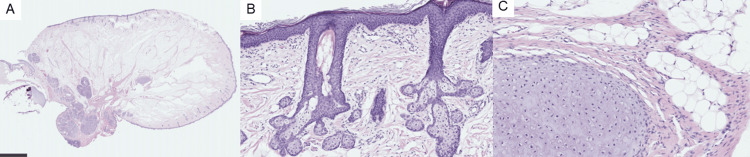
Histopathological findings of the hairy polyp (A) The polypoid tumor is covered with squamous epithelium, skin dermis, and adipose tissues. (B) Hair follicles and sebaceous glands are observed in the skin. (C) Cartilage tissues are observed in the subcutaneous tissues.

The tumor’s base was between the anterior and posterior palatine arches. Surgical resection was performed perorally. The diameter of the resected polyp was 1.5 cm. Histopathological examination revealed that the tumor was covered with squamous epithelium and that skin appendages (such as hair follicles and sebaceous glands), adipose tissues, and cartilages were beneath the epithelium, which confirmed the diagnosis of a hairy polyp (Figure 2).

The patient was followed up for three years after the surgery, and recurrence of the hairy polyp was not observed.

## Discussion

The World Health Organization’s classification of head and neck tumors describes that hairy polyp is considered to be likely developmental abnormalities of the first or second branchial cleft [3]. Although there have been reports of hairy polyps in older children, most are found during the neonatal and infant stages. The diagnosis of hairy polyp is histologically confirmed by a layer of stratified squamous epithelium and dermis with keratinizing, beneath which is composed of adipose tissue with admixed fibrous tissue and may also contain mature cartilage and bone.

Among cases of hairy polyps reported over the past 25 years, the most common site was the lateral nasopharynx (29.5%), followed by the tonsils (17.9%) [1]. The most common complaint at the time of admission was dyspnea (50%), followed by dysphagia (24.6%) [1]. While it is easy to recognize cases where the tumor protrudes outside the oral cavity [2], there are also cases where the presence of the tumor is difficult to recognize from the outside, so it is important to examine the nasopharynx in neonates and infants with respiratory disorders. It has been reported that hairy polyps <3 cm in diameter is often overlooked during oral examinations. However, respiratory distress and cardiac arrest are more common in these polyps than in those measuring >3 cm [4].

Approximately 10% of patients with hairy polyps reportedly have a cleft palate, which may be related to various arch deformities such as cleft palate, absence of the uvula or external auditory canal, and facial hypoplasia [5]. Our case also had a cleft palate, suggesting that cleft palate may be related to the occurrence of hairy polyps.

## Conclusions

Hairy polyps should be considered a cause of respiratory distress in neonates and infants, especially those with a cleft palate. Because hairy polyps can be fatal, if a mass is found in the pharynx, it is important to perform appropriate respiratory management and proceed to surgery safely.
